# Investigation of 13q14.3 deletion by cytogenetic analysis and FISH technique and miRNA-15a and miRNA-16-1 by real time PCR in chronic lymphocytic leukemia

**DOI:** 10.4314/ahs.v22i3.20

**Published:** 2022-09

**Authors:** Melike Yılmaz, R Dilhan Kuru, Isil Erdoğan, Teoman Soysal, Seniha Hacıhanefioglu, Onur Baykara

**Affiliations:** 1 Istanbul University Institute of Experimental Medical Research, Genetic Department, Istanbul, Turkey; 2 Istanbul University- Cerrahpasa, Cerrahpasa Medical Faculty, Medical Biology Department, Istanbul, Turkey; 3 Istanbul University -Cerrahpasa, Cerrahpasa Medical Faculty, Department of Internal Medicine, Division of Hematology, Istanbul, Turkey

**Keywords:** CLL, miR-15a/miR-16-1, 13q14.3 deletion

## Abstract

**Background:**

The most frequent cytogenetic aberration is 13q14.3 deletion in Chronic Lymphocytic Leukemia (CLL). HsamiR-15a/hsa-miR-16-1 are tumor suppressor miRNAs encoded from 13q14.3 region.

**Objectives:**

The aim of this study was to investigate the 13q14.3 deletion using molecular and cytogenetic techniques and association with miRNA-15a/miRNA-16-1.

**Materials And Methods:**

We used peripheral blood samples of 30 CLL patients who were either induced and or non-induced with DSP30+IL-2 to determine 13q14.3 deletion by karyotyping and iFISH. Expression levels of hsa-miR-15a/miR-16-1 were measured using qRT PCR and compared with deletions.

**Results:**

13q14.3 deletion was detected in 8.6% of cases by karyotyping and in 65% by iFISH. Mosaic forms (monoallelic+biallelic) were observed in 50% of cases. Besides determining common chromosome abnormalities such as add(2)(q37), t(2;7) (p11.2;q22), del(6)(q13q21), del(6)(q25), add(9)(q21), del(11)(q23), t(11;14)(q13;q32), del(13)(q11q12), del(13)(q12q14), add(14) (q23), del(14)(q23), t(14;19)(q32;q13.1), del(15)(q23), del(17)(p12), t(18;22)(q21;q11.2), add(21)(p13) and t(17;21)(q11.2;122), we also determined t(1;13)(q32;q34), inv(2)(p25q21), del(13)(q22q32), t(14;19)(q24;q13), dup(17)(q21q23), der(21;21)(p13;p13) which have not been reported previously. Mitotic index data was found statistically significant and DSP30+IL-2 increased mitotic index by 2.5 folds. Association between decreased miR-16-1 expression and deletions was statistically significant.

**Conclusion:**

We suggest that cytogenetic and iFISH analyses are complementary and use of DSP30+IL-2 is effective .in CLL. Decreased expression of hsa-miR-16-1 is remarkable.

## Introduction

Chronic Lymphocytic Leukemia (CLL) is characterized by the gradual accumulation of functionally immature, small, monoclonal CD5 + and CD23 + B cells most of which are nonproliferating cells arrested at G0/G1 phase of the cell cycle 1. CLL comprises about 30% of all cases of adult leukemia in Western World affecting both sexes and chromosomal abnormalities are widely used in CLL to prognose, treat, and follow the overall survival in patients^2^. These abnormalities include del(13)(q14), del(11) (q22), trisomy 12, and del(17)(p13) and they can be detected using conventional banding techniques. The most frequent abnormality is deletion of 13q14 which is associated with a favorable prognosis 3,4.

Deletion of 13q14 is mostly monoallelic (in 76% of cases), but also it is detected in biallelic (24%) and mosaic forms by iFISH 4,5.

Conventional karyotyping and Interphase Fluorescence in Situ Hybridization (iFISH) techniques are widely used to detect cytogenetic abnormalities seen in CLL. However, approximately 50% of patients with CLL can be analyzed by conventional karyotyping due to the very low responsiveness of CLL cells to mitogenic stimuli in vitro^3^. Recently a new mitogen CpG-oligodinucleotide (CpD-ODN) called DSP30 was used in CLL cytogenetics which was reported to be the most effective CpG-ODN in stimulating human B cells 6.

MicroRNAs (miRNAs) are 20–22 nucleotide long small noncoding RNAs that can bind to untranslated regions (UTRs) of target mRNAs resulting in translational repression or mRNA degradation 7. DLEU2 gene encoding miR-15a and miR-16-1 are located within the deletion region of 13q14. Both miR-15a and miR-16-1 have been shown to exhibit tumor-suppressing activities by inducing apoptosis and inhibiting proliferation. Generally speaking, levels of both miRNAs are decreased due to deletion 13q14 but the physical loss of 13q14 is not the only mechanism causing a decrease in miRNAs levels. Epigenetic regulations and defects in miRNA biogenesis can contribute to their dysregulation as well^8^,^9^. In this study, we investigated miR-15a and miR-16-1 levels by qRT-PCR and compared with 13q14.3 deletion detected by conventional karyotyping and iFISH techniques. We also investigated other possible cytogenetic abnormalities and effects of DSP30 + IL-2 combination using conventional karyotyping techniques in patients with CLL.

## Materials And Methods

Peripheral blood was collected from 30 chronic lymphocytic leukemia patients. All patients were followed up in division of hematology. 18 (60%) of patients are males and 12 (40%) are females. 15 healthy control subjects (9 (60%) males and 6 (40%) females) were also included in the study. Expression levels of miR-15a and miR-16-1 were measured using qRT-PCR, while deletion of 13q14 region was assessed using conventional cytogenetic and iFISH techniques. Informed consent was obtained from mall patients prior to the study and all study was conducted following Declaration of Helsinki, 2013. The Ethical Committee of Istanbul University-Cerrahpasa, Cerrahpasa Medical Faculty approved the study (nr:83045809/604/0101/114304).

Conventional Cytogenetic Analysis Using DSP30 + IL-2 Conventional karyotyping analysis was performed in all patients using standard protocols. Peripheral blood samples (∼1 ml) of patients were added in three separate conical tubes and suspended in 2.5 ml of 1640 RPMI (Biochrome, Berlin, Germany). We added 10 µl of CpG-ODN DSP30 (TıbMolBiol, Berlin, Germany) with a concentration of 1µM and 50 µl IL-2 (Roche, Mannheim, Germany) with a concentration of 100 U/mL for both first and second tubes. Third tubes were used as control which do not include DSP30 and IL-2. All samples were cultured at 37oC for 72 h. 50 µl of colcemid (KaryoMax, Thermo Fisher Scientific, Waltham, MA, USA) (with a final concentration of 10 µg/ml) was added prior to harvesting of the cells.

Chromosome preparation and staining using G-Banding technique was performed following standard procedures in patients and control samples, as described elsewhere^10^. Karyotypes were scored according to the International System for Human Cytogenetic Nomenclature (ISCN) 2016 11.

Mitotic index was scored by counting metaphases on a slide containing 1000 cells per mitosis in induced by DSP30 and non-induced patient samples and compared to the controls.

### iFISH Experiments

iFISH analyses were performed in interphase cells using 13q14.3 locus specific probe (Cytocell LSI-D13S319 Plus, Cambridge, UK) in induced samples by DSP30 of all patients. Cultured samples were re-suspended with fresh Carnoy fixative (3 : 1 methanol : acetic acid) for two hours at room temperature (RT) , centrifuged, removed the supernatant, and then re-suspended in fresh fixative for a few more minutes before spreading and then Cytocell iFISH protocol was performed 12. Three hundred interphase nuclei were scored in all samples. Same protocols were applied for the control samples.

### qRT-PCR Experiments

miRNA was isolated using Exiqon miRCURY RNA isolation kit (Exiqon A/S, Vedbaek, Denmark) following the manufacturer's instructions and cDNA was synthesized using miRCURY LNA™ microRNA PCR, Polyadenylation and cDNA synthesis kit II (Exiqon A/S, Denmark) with an initial amount of 10 ng of RNA. cDNA synthesis was performed in T100 Thermal Cycler System (Bio-rad Laboratories, Hercules, CA, USA) with following conditions: 42°C for 60 min, 95°C for 5 min and 4 °C for cooling. The expression levels of miR-15a and miR-16-1 were determined with a quantitative system based on SYBR Green probe technology [miRCURY LNA™ microRNA PCR, ExiLENT SYBR® Green master mix (Exiqon S/A, Denmark)]. The assay was performed in a total volume of 10 µl and contained 5 µl 1xPCR master mix, 1 µl of each primer (miRCURY LNA PCR Primer mix for hsa-miR-15a-5p and hsa-miR-16-1-3p; Exiqon S/A, Denmark) and 4 µl of cDNA sample. The expression levels of miRNA-15a and miR-16-1 were measured in Light Cycler 1.5 System (Roche Diagnostics, Mannheim, Germany) under the following conditions: 95°C for 10 min initial denaturation, 40 cycles of 95°C 10 s and 60°C for 1 min for denaturation, 1 cycle of 95°C for 1min, 40°C for 2 min, 95°C for 1s for melting. Relative miRNA expression levels were calculated using the 2- ΔΔCT method 13. The U6 housekeeping was used as a reference. All experiments were performed in triplicates.

### Statistical Analysis

Statistical analyses were performed with the SPSS 21 software (IBM Corp. Released 2012. IBM SPSS Statistics for Windows, Version 21.0. Armonk, NY: IBM Corp). Wilcoxon test was used to confirmation of mitotic index in DSP30 + IL-2 induced and non-induced samples and mitotic index differences between patient and control samples (p<0.001). Cut-off values of deletion types in patients and control samples were calculated using ROC analysis. Association between miRNAs (hsa-miR-15a, hsa-miR-16-1) expression levels and 13q14.3 deletion types were analyzed using Mann-Whitney U test. Significance value was determined as (p < 0.05).

## Results

**Figure 1:**
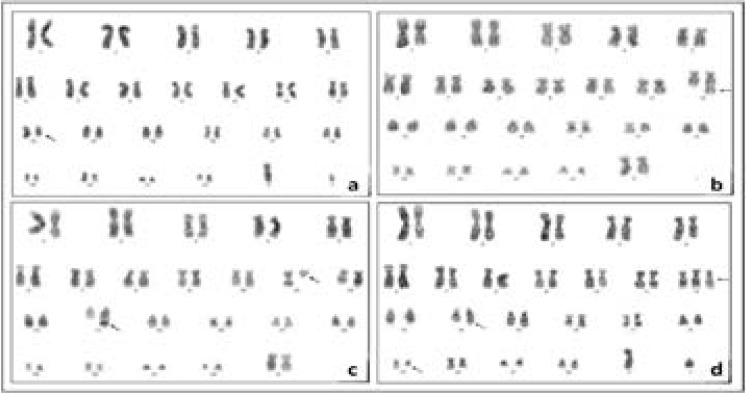
Karyogram samples including some chromosomal abnormalities in different patients 46,XY,del(13)(q14.3) (a), 46,XX,dup(12)(q13q24) (b), 46,XX,t(11;14)(q13;q32) (c), 46,XY,+12,t(14;19)(q32;q13.1) (d)

In DSP30 + IL-2 induced samples, a sufficient number of metaphases suitable for cytogenetics analysis was obtained in 23/30 (76.6%) of the patients and clonal chromosomal aberration was detected in 16/23 (69.5%) of the patients. A normal karyotype was found in 7/23 (30.4%) of the patients. 13q14.3 deletion was detected only in 2/23 (8.6%) of the patients with conventional karyotyping techniques. In DSP30 + IL-2 non-induced samples metaphases suitable for cytogenetics analysis were observed in 7/30 (23.3%) of the patients and chromosomal aberrations were detected in 2/7 (28.5%) of the patients. 13q14.3 deletions were not observed in non-induced samples by conventional karyotyping. Cytogenetic aberrations of twenty-three successfully karyotyped patients are presented in Table 1. Also some different abnormalities we found in induced samples are shown in Figure 1.

**Table 1:** Conventional cytogenetic results in samples induced and non-induced with DSP30 + IL-2 (-): No results are available due to absent cells and/or low mitotic index

Case No	DSP30+IL-2 induced	DSP30+IL-2 non-induced
1	-	-
2	-	-
3	46,XY[22]	-
4	46,XX[14]	-
5	41∼46,XY,-Y[3],t(2;7)(p11.2;q22)[28],del(6)(q?q?) [9],der(17)t(3;17)(q21;p11.2)[29],-21[5], +21[5],add(21)(p13)[6],der(21;21)(p13;p13)[14], mar1[3][cp31],/46,XY[2]	-
6	46,XY,del(17)(p12),dup(17)(q21q23)[3]/46∼47,XY, +13,del(13)(q11q12)[cp2] /46,XY[4]	46,XY[4]
7	46,XX,der(13)(q12q14)del(3)(q22q32)[8]/38∼46,XX, del(6)(q?q?)[12],der(13)del(13)(q12q14) del(13)(q22q32)[16],-17[4],t(17;21)(q11.2;q22)[2], der(21)t(17;21)(q11.2;q22)[2],-21[3],-22[3][cp19] /46,XX[2]	-
8	40∼45,XY,-20[cp4]/46,XY[19]	46,XY[3]
9	46,XY[8]	-
10	-	-
11	43∼49,XY,+8[1],del(14)(q23)[21][cp21]/92∼98,XXY, +4[2]+8[1],+14[2]del(14)(q23)x2[4],+19[2][cp4] /46,XY[2]	45,XY,del(14)(q23)[cp1]/46,XY[1]
12	46,XY,t(1;13)(q32;q34)[3],del(6)(q13q21)[2][cp3]/ 46,XY[8]	-
13	46,XY[19]	46,XY[11]
14	-	-
15	42∼46,XX,-10[3],t(11;14)(q13;q32)[10],-21[3][cp12] /46,XX[9]	-
16	46,XX,del(11)(q23)[2]/46,XX[5]	-
17	39∼46,XY,t(18;22)(q21;q11.2)[11],-21[3][cp11] /46,XY[5]	-
18	-	-
19	47,XY,+12[22],add(14)(q32)[3],t(14;19)(q32;q13.1) [15],t(14;19)(q24;q13)[2][cp22]	43∼48,XY,+12[10],t(14;19)(q32;q13.1)[4], add(14)(q32)[3],t(14;19)(q24;q13)[1][cp10]
20	41∼46,XY,+12[20],-21[3][cp21]/46,XY[2]	-
21	46,XX,dup(12)(q13q24)[2]/46,XX[24]	-
22	46,XX,add(2)(q37),del(6)(q25),del(15)(q23),+mar1[2]/ 46,XX,inv(2)(p25q21)[cp2]/46,XX[40]	46,XY[1]
23	46,XX[10]	-
24	42∼45,XX,-21[cp4]/46,XX[16]	-
25	46,XY[16]	-
26	46,XX,+20[cp2]/46,XX[8]	-
27	46,XX[2]	-
28	-	-
29	-	-
30	46,XY,del(13)(q12q14)[10]/46,XY[7]	-

**Figure 2:**
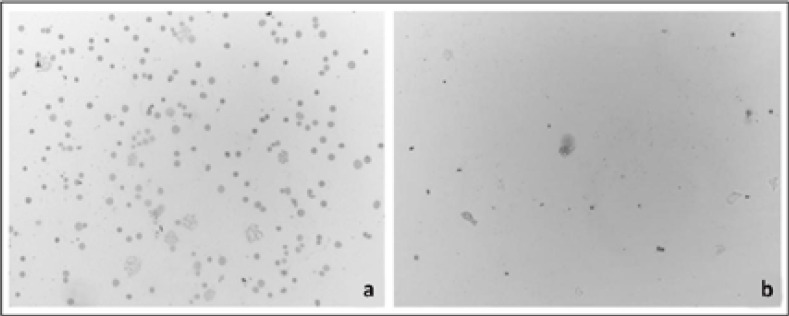
Images of metaphase plaques and cell density in different patients samples with DSP30 + IL-2 (a), without DSP30 + IL-2 (b) (10x magnification)

Mitotic index was calculated as 10.48% in DSP30 + IL-2 induced samples and as 4.2% in noninduced samples whereas defıned as %45 in control subjects. We identified that using DSP30 increased mitotic index by 2.5 folds. The difference in induced and non-induced samples was found statistically significant (p < 0.001) (Table 2). Metaphase plaques (number of metaphases) and cell density in induced and non-induced samples are shown in Figure 2.

**Table 2:** Results of Mann-Whitney Test Statistic used for compare of mitotic index with using and non-using DSP30 + IL-2 samples

	Induced with DSP30 + IL-2	Non-induced with DSP30 + IL-2	P value
Patient Median (min-max)	3.5 (0–46.8)	0 (0–2.50)	< 0.001

13q14.3 deletion was detected in 2/23 (8.6%) of DSP30 + IL-2 induced patients by conventional karyotyping whereas this deletion was detected by iFISH in 17/26 (65.3%) of the same patients. Monoallelic and mosaic deletions were determined in 17/26 (65.3%) and in 13/26 (50%) of the patients, respectively. Statistically; cut-off values, performed by ROC analyses (p < 0.05) (Table 3), were calculated as 1.88% and 0.13% for monoallelic (2G/1R) and biallelic (2G) deletions, respectively.

**Table 3:** Results of ROC analysis for del(13q14.3)

Test result Variable(s) (%)	Area	Std. Error	p value	%95 Confidence Interval
2G/2R (Normal)	0.314	0.077	0.014	0.164–0.465
2G/1R (Monoallelic D.)	0.674	0.076	0.022	0.523–0.823
1G/1R (Monosomy)	0.606	0.078	0.163	0.452–0.759
2G (Biallelic D.)	0.737	0.069	0.002	0.601–0.873

**Figure 3:**
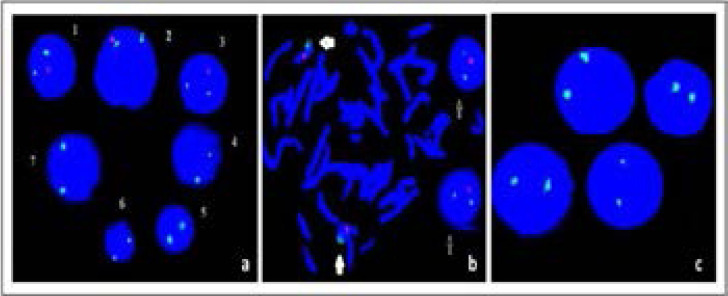
FISH images of monoallelic and biallelic deletions of 13q14.3 in different patient samples induced with DSP30 + IL-2: iFISH images of monoallelic (1, 2, 3) and biallelic (4, 5, 6, 7) 13q14.3 deletions (a), Metaphase FISH (mFISH) images of normal cell and iFISH images of monoallelic deletion cells (b), iFISH images of biallelic 13q14.3 deletions cells (Green signal:Control = 13qter/13q34 / Red signal: Deletion = 13q14) (c)

**Figure 4:**
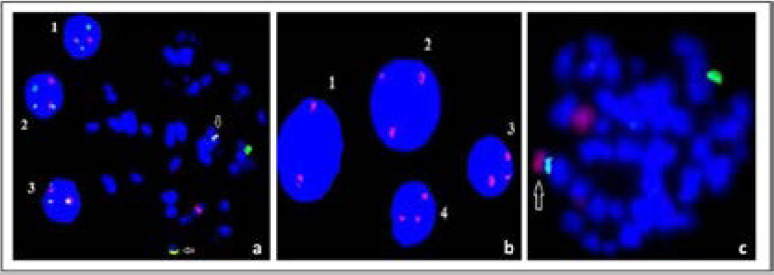
FISH images of different chromosomal abnormalites in DSP30 + IL-2 induced samples: iFISH(1,2,3) and mFISH images of t(11;14)(q13.3;q32.33) (Red Signal,11q13.3; Green Signal,14q32.33), (Yellow Signal, Fusion (t(11;14)(q13.3;q32.33)) (a), iFISH images of normal (1) and trisomy 12 (2,3,4) cells (Red Signal, Centromere: 12/12p11.1-q11.1/D12Z3) (b), mFISH image of der(21)t(17;21) (Red signal, chr.17; Green Signal, chr. 21, arrow:fusion chromosome) (c).

iFISH images of mono and biallelic deletions at 13q14 and other chromosomal abnormalities except for 13q14 that were detected in DSP30 + IL-2 induced samples are shown in Figure 3 and Figure 4, respectively.

We showed that miRNA-15a expression was decreased in 13/30 (45.5%), increased in 15/30 (50%), not changed in 2/30 (6.6%) of the patients whereas miR-16-1 expression decreased in 15/30 (50%), increased in 14/30 (46.6%), not changed in 1/30 (3.3%) of the patients when compared to controls. Expression levels of both miRNAs were simultaneously decreased in 7/30 (23.3%) and increased in 8/30 (26.6%) of the patients. Hsa-miR-15a and hsa-miR-16-1 expression changes are shown in Table 4. Statistical analyses showed that changes were not significantly correlated with deletions of 13q14 (Table 5) but decreased miRNA-16-1 expressions were found to be correlated (Table 6).

**Table 5:** Mann-Whitney U test P values showing the association between the expression levels of hsa-miR-15a gene and 13q14.3 deletions

(hsa-miR-15a)				
	2G/2R	2G/1R	1G/1R	2G
P value	0.913	0.477	0.701	0.542

**Table 6:** Mann-Whitney U test P values showing association between expression levels of hsa-miR-16-1a gene and 13q14.3 deletions

(hsa-miR-16-1)				
	2G/2R	2G/1R	1G/1R	2G
P value	0.017	0.008	0.585	0.007

## Discussion

CLL is a blood and bone marrow disease developing slowly over time resulting with over production of lymphocytes by bone marrow mostly affecting older adults. Many genetic and epigenetic factors contribute to the formation of the disease 14–16. Therefore, detecting molecular and cytogenetic abnormalities has vital importance in diagnosis and treatment of the disease 17,18. However, it is extremely difficult to diagnose the abnormalities using only conventional karyotyping techniques due to the low mitotic index of lymphocytes. Stimulating the CLL cells using various agents for cytogenetic analysis has major importance in diagnosis. One of these effective immunostimulatory agents is DSP30. Recent studies have shown the efficacy of DSP30 on CLL cells either alone or with another agent (IL-2) 17–22.

In this study, we used conventional karyotyping, iFISH, and qRT-PCR techniques in 30 CLL patients and 15 healthy controls to detect the abnormalities developed in CLL. As karyotyping is considered as a low efficient method due to the low proliferation of CLL cells, iFISH technique and DSP30 have found a wider area of application to stimulate CLL lymphocytes in order to detect chromosomal abnormalities.

Regarding the chromosome banding, we found that clonal chromosomal abnormalities were present in 16/23 (69.5%) patient samples induced with DSP30 + IL-2 using the conventional karyotyping method. The occurrence of del(13q), del(11q), +12, and del(17p) was established in 8.6% (2/23), 4.3% (1/23), 8.6% (2/23) and 4.3%(1/23), respectively. These values are comparable to the data published so far 18,23,24. Other chromosomal abnormalities we determined are shown in Table 1 and common abnormalities diagnosed in CLL causing bad prognosis such as t^11^;^14^(q13;q32) 25–27, dup^12^(q13q22), t^14^;^19^(q32;q13.1) accompanying with +12 and add^14^(q32) 26–28 were also present in our study.

Trisomy 12 mechanism and target genes are not clear in CLL and associate with t(14;19)(q32;q13). So; previous studies and our results support that these three abnormalities can be clonal changes in leukemogenesis of CLL and investigating this mechanism can be beneficial in order to understand the pathogenesis of CLL and develop new treatment options. We also determined t(1;13) (q32;q34),inv(2)(p25q21),t(3;17)(q21;p11.2),del(13) (q22q32), t(14;19)(q24;q13), dup(17)(q21q23), der(21;21) (p13;p13) which have not been reported previously. To our knowledge, this is the first study reporting t(14;19) (q24;q13) in CLL. Previous studies have reported t(14;19) (q32;q13.1) in CLL. However, our new translocation spans a larger area on the chromosome starting from q24 region. This large area may be associated with CLL. Balanced rearrangements are rare in CLL and we detected balanced translocation rob^21^;^21^p^13^;p^13^.

In non-induced samples; we determined successful karyotyping in 6/30 (20%) of the patients and detected clonal chromosomal abnormalities in 2/6 (33%) of the patients. However, no chromosomal abnormalities were detected in 4 induced samples. Also, we determined that DSP30 + IL-2 caused an increase in the mitotic index by 2.5-folds. So our results showed that using DSP30 + IL-2 in CLL patients is very effective to increase mitotic index which allows us to detect sufficient number and quality metaphases and detect chromosomal abnormalities by inducing division of leukemic cells as reported in other studies 17–20.

We investigated 13q14.3 deletion with both conventional karyotyping and iFISH techniques. del(13q) was detected only in 8% of patients with karyotyping while iFISH revealed it in 65.3%. Previous studies have reported rates varying between %1-56 18,23,29,30. Similar to previous studies, our results indicate that iFISH technique is a very sensitive and specific method for routine diagnosis of CLL. In order to detect the abnormalities seen in chromosomes, conventional karyotyping is the most common method. On the other hand, it is obligatory to know the abnormality in advance to apply iFISH method which uses specially designed probes. Use of both methods at the same time can yield better results. In conclusion, DSP30 + IL-2 should be preferred for routine conventional karyotyping of CLL and both techniques should be complementarily applied.

Our study showed decreased expression of miR-16-1 which correlates with monoallelic and biallelic deletion.

Recent studies reported decreased expression of miR-15a and miR-16-1 in CLL cases. Calin et al. reported decreased miR expression in their study^16^. Smonskey et al. showed that the decrease in miR-15a expression correlated with deletions but no significant association between miR-16-1 expression and deletions was found in their study^9^.

The main limitation of our study is small sample size. However, this did not affect the methodology or interpretation of the results. Nevertheless, it would be plausible to study a larger group of patients. In addition, other miRNAs need to be studied to understand the effect of these special molecules on disease progression.

In conclusion, we didn't find any correlation between miR-15a expression levels and deletions but some patients with del(13q) had increased miRNA expression levels or some patients without del(13q) decreased expression levels. miR-16-1 expression was found to be decreased but it was not related to deletions. So these findings can suppose that expression of both miRNAs not only regulates chromosomal loss but also other mechanisms such as epigenetic changes. Also, increased expression levels of both miRs in del(13q) positive patients may be due to the presence of homologous gene loci miR-15b/miR-16-2 in the 3q25-26.1. Our results indicate that miR16-1 plays an effective role in CLL pathogenesis when compared with miR15a.

According to our results, FISH method is more sensitive to detect deletion of 13q14.3 than conventional karyotyping methods. We also suggest that using DSP30 + IL-2 combination can be very useful for karyotyping methods of CLL. Also miR-16-1 expression levels can be used to investigate in CLL patients with del(13q).
